# Potential Role of Rhizobacteria Isolated from Citrus Rhizosphere for Biological Control of Citrus Dry Root Rot

**DOI:** 10.3390/plants10050872

**Published:** 2021-04-26

**Authors:** Said Ezrari, Oumayma Mhidra, Nabil Radouane, Abdessalem Tahiri, Giancarlo Polizzi, Abderrahim Lazraq, Rachid Lahlali

**Affiliations:** 1 Phytopathology Unit, Department of Plant Protection, Ecole Nationale d’Agriculture de Meknès, Km10, Rte Haj Kaddour, BP S/40, Meknès 50001, Morocco; Said.ezrari@usmba.ac.ma (S.E.); oumayma.mhidra.1996@gmail.com (O.M.); Nabil.radouane@usmba.ac.ma (N.R.); atahiri@enameknes.ac.ma (A.T.); 2 Laboratory of Functional Ecology and Engineering Environment, Department of Biology, Sidi Mohamed Ben Abdellah University, P.O. Box 2202, Route d’Imouzzer, Fez 30000, Morocco; abderrahim.lazraq@usmba.ac.ma; 3 Dipartimento di Agricoltura, Alimentazione e Ambiente, sez. Patologia Vegetale, University of Catania, Via S. Sofia 100, 95123 Catania, Italy

**Keywords:** Citrus, *Neocosmospora solani*, Dry root rot, biological control, PGPR

## Abstract

Citrus trees face threats from several diseases that affect its production, in particular dry root rot (DRR). DRR is a multifactorial disease mainly attributed to *Neocosmospora* (*Fusarium*) *solani* and other several species of *Neocosmospora* and *Fusarium* spp. Nowadays, biological control holds a promising control strategy that showed its great potential as a reliable eco-friendly method for managing DRR disease. In the present study, antagonist rhizobacteria isolates were screened based on in vitro dual culture bioassay with *N. solani*. Out of 210 bacterial isolates collected from citrus rhizosphere, twenty isolates were selected and identified to the species level based on the 16S rRNA gene. Molecular identification based on 16S rRNA gene revealed nine species belonging to *Bacillus*, *Stenotrophomonas*, and *Sphingobacterium* genus. In addition, their possible mechanisms involved in biocontrol and plant growth promoting traits were also investigated. Results showed that pectinase, cellulose, and chitinase were produced by eighteen, sixteen, and eight bacterial isolates, respectively. All twenty isolates were able to produce amylase and protease, only four isolates produced hydrogen cyanide, fourteen isolates have solubilized tricalcium phosphate, and ten had the ability to produce indole-3-acetic acid (IAA). Surprisingly, antagonist bacteria differed substantially in their ability to produce antimicrobial substances such as bacillomycin (five isolates), iturin (ten isolates), fengycin (six isolates), surfactin (fourteen isolates), and bacteriocin (subtilosin A (six isolates)). Regarding the PGPR capabilities, an increase in the growth of the bacterial treated canola plants, used as a model plant, was observed. Interestingly, both bacterial isolates *Bacillus subtilis* K4-4 and GH3-8 appear to be more promising as biocontrol agents, since they completely suppressed the disease in greenhouse trials. Moreover, these antagonist bacteria could be used as bio-fertilizer for sustainable agriculture.

## 1. Introduction

Citrus is an important economic crop in Morocco, covering an area of 126.600 ha with a production of 2.6 million tons of citrus fruits a year. The annual exportation of citrus fruits to Europe and other western countries has reached 755.000 tons [1,2]. However, citrus plantations are threatened by several pathogens of which the *Fusarium* species, mainly *F. solani*, is causing citrus dry root rot (DRR) disease, which is responsible for significant economic loss [1,3,4]. In addition, *Fusarium* species can cause other serious diseases on citrus plantation such as twig rot, decline dieback, twig blight, and vascular wilt, thereby are a major threat to citrus production worldwide [1,5,6]. *Fusarium sensu latu* was recently segregated into several *Fusarium*-like genera (i.e., *Bisifusarium* [*Fusarium dimerum* specie complex (SC)], *Neocosmospora* [*Fusarium solani* SC] and *Rectifusarium* [*Fusarium ventricosum* SC] [7]. DRR symptoms are characterized by suddenly wilt and fall of the tree. The pathogen penetrates citrus roots and cause root rot; the roots become blackened with discoloured peels and necrosis, and when the pathogen reaches the xylem vessels it leads to the weakening of the tree. The disease worsens with biotic (*Phytophthora* spp., *citrus tristeza virus* (CTV), or attacks by pests such as nematodes (e.g., *Tylenchulus semipenetrans* Cobb), rodents, and insects) and/or abiotic (drought, root asphyxiation due to over-watering, high temperature, poor root aeration, and excessive fertilization) stressors [1,8,9,10].

DRR disease is increasingly becoming an important threat to citrus plantations worldwide [1,3,4,8,11]. However, no curative control strategy is currently available to suppress this disease. The common strategy used for the disease control is mainly based on an integrated management approach combining sanitation measures, cultural practices, the use of chemical control and tolerant cultivars [1,8]. Information on the use of alternative approaches against DRR is sparse [12,13]. To date, there is not an effective control method capable of controlling these soil-borne pathogens. Therefore, an integrated protection approach becomes essential to overcome this problem [14]. However, in the last two decades, biological control using biocontrol agents (BCAs) was highly studied to be used as an effective strategy for the control of diseases that affect crops [15].

Nowadays, the biological approaches for improving crop production are gaining more attention, especially from producers, agronomists, and environmental scientists. Accordingly, an extensive and rigorous studies are taking place worldwide to explore and deepen the wide range of bacteria in the rhizosphere having novel traits in favour of the plant growth and protection [16,17]. Rhizosphere are colonised by a diversity of complex bacteria, which can play a central role for plant health and growth [18]. Plant promoting growth rhizobacteria (PGPR) can act as phytostimulators, phytoprotectors, biopesticides, or rhizoremediators, and biofertilizers by producing several plant growth regulators that contribute to the plant's growth process such as cell division, root extension, cell enlargement, and the induction of germination and facilitating nutrient uptake [19,20,21]. Biological control is a control strategy based on the screening of BCAs from the host-microbiome or from area which closely correspond to the climate and the type of soil of the infected region in order to identify and stimulate the antagonists naturally present in this area [22,23].

The biological control of diseases caused by *Fusarium* species has proven to be one of the most effective eco-friendly strategies [22,24,25]. BCAs combine several mechanisms to exert their effect. The control method used is based on direct mechanisms by inhibiting the pathogenic agent via the secretion of antibiotic substances or by indirect mechanisms, namely competition for common trophic and/or spatial resources such as competition for one or more nutrient resources (such as nitrogen, carbon, and/or iron), acquisition of nutrients for plants (solubilization of phosphorus (P), and nitrogen fixation (N), production of phytohormones, and volatile compounds) [26,27,28]. In the case of antibiosis, BCAs act by secreting microbial volatile compounds such as hydrocyanic acid (HCN) and non-volatile like diffusible non-volatile metabolites, secondary metabolites, antibiotics, and lytic enzymes mainly cellulase, chitinase, biosurfactants (lipopeptides and bacteriocins) which are capable of poisoning the pathogen as well as reducing or even inhibiting its mycelial growth [29,30].

Lipopeptides are cyclic amphiphilic oligopeptides with a low molecular weight produced by non-ribosomal peptide synthetase [31] with strong antimicrobial activities [32,33] due to their capacity to interact with the cell membrane resulting in the formation of pores and the solubilization of the membrane (at higher concentrations) [34]. Surfactins /lichenysins, iturins/bacillomycins/mycosubtilins, and fengycins/plipastatins are the three main families of lipopeptides [35]. The bacteriocins, including subtilosin A, are ribosomally produced, with potent antimicrobial peptides, by diverse groups of micro-organisms able to form pores in the membrane [35,36].

Studies on the biological control of citrus DRR disease are almost absent worldwide, which might urge the exploration of the core microbiome of citrus rhizosphere for this purpose. The rhizosphere is an environment with high microbial diversity, and it is known to be a reservoir of microorganism that possess some important biological activities [37]. In addition, microorganisms that are present in the rhizosphere of healthy citrus trees may have the possibility to compete for nutrients, improve plant growth, and improve their abilities to resist to different biotic and abiotic stress. Therefore, the present study aimed at exploring the potential biocontrol of bacterial isolates, from the citrus rhizosphere of different Moroccan regions, against *Neocosmospora* (*Fusarium*) *solani*, the most dominant species associated with DRR disease. Furthermore, the most effective bacterial isolates selected from dual culture bioassay will be characterized to the species level using 16S rDNA gene. These bacteria will be screened for (i) biochemical traits and genes involved in production of antifungal compounds, (ii) their PGPR capabilities with canola (*Brassica napus*), and (iii) *in planta* greenhouse trials to control DRR disease.

## 2. Results

### 2.1. Antagonistic Activity

**Figure 1:**
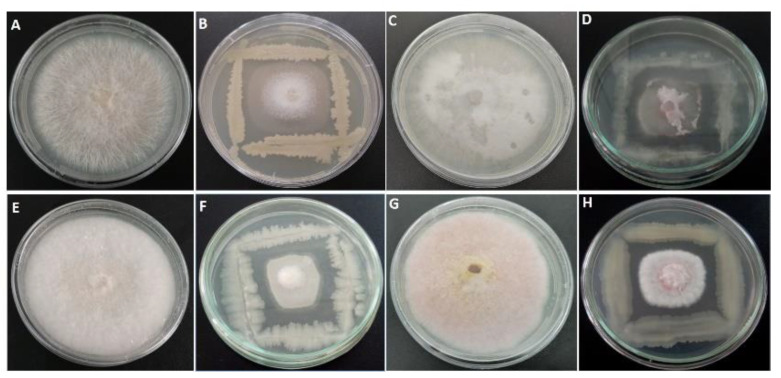
In vitro dual cultures showing antagonistic activity of bacterial isolates against four isolates of *Neocosmospora solani* and *Fusarium* spp. on PDA medium at 25 °C for 7 days. Control (**A**) and representative bacterial isolate (GH3-8) (**B**) against *N. solani*. Control (**C**) and representative bacterial isolate bacteria (GH1-2) (**D**) against *F. oxysporum*. Control (**E**) and representative bacterial isolate (BM1-3) (**F**) against *F. equiseti*. Control (**G**) and representative bacterial isolate (BM4-3) (**H**) against *F. brachygibbosum*.

A total of 210 morphologically different bacterial colonies isolated from different citrus rhizosphere areas across the country were tested for their antagonistic activity against *N. solani* using dual culture bioassay (Figure 1). The zone of inhibition offered by each bacterium was assessed 7 days post-incubation period (Table 1). Results underlined that twenty isolates displayed an important antifungal activity against *N. solani* (>60%) with very highly significant difference. The selected antagonistic bacteria were also tested for their antifungal activity against *Fusarium oxysporum*, *F. brachygibbosum*, and *F. equiseti* (Table 1). The most promising bacterial isolates were k4-4, TD1, and GH3-8 with inhibition rates of mycelial growth of 72.22, 71.50, and 68.93%, respectively.

### 2.2. Effect of Bacterial Isolates on the Mycelial Structure of Neocosmospora Solani

**Figure 2:**
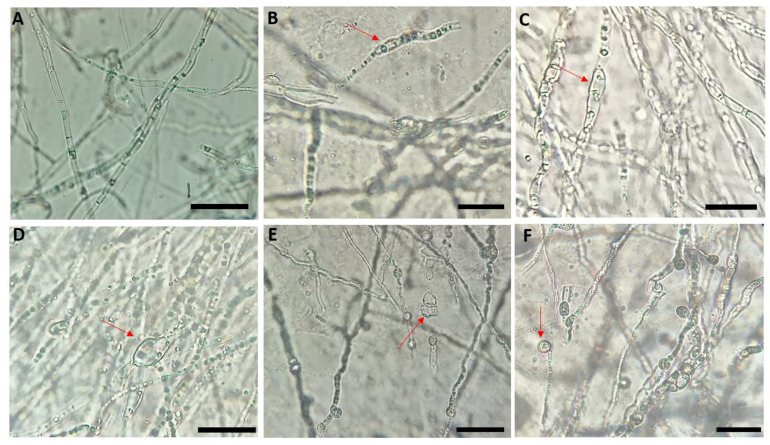
Microscopic observation (×40) of the mycelial structure of *Neocosmospora solani* in co-culture with the antagonist bacteria. Control hyphae displaying equal widths and even surfaces with active branching and hyphae from dual culture displayed damaged morphology and substantial abnormalities: hyphal deformation with several distorted and condensed structures with large vacuole and massive conglobated along hyphae with uneven surface. (**A**) Untreated control, (**B**) K4-3, (**C**) TD1, (**D**) B2-1, (**E**) BM1-3, and (**F**) GH3-8. Scale bar = 50 µm.

Microscopic observations of the mycelium of *N. solani* in co-culture with the antagonist bacteria displayed damaged morphology and distinct cytological alterations when compared to the untreated control (Figure 2A). In most cases, the alterations correspond to hyphal swelling, deformation, and vacuolation of the mycelial structure (Figure 2B–D), and massive conglobated along hyphae with uneven surface (Figure 2E,F) and sometimes associated with a degradation of the mycelium and a release of the cytoplasmic contents (Figure 2E).

### 2.3. Identification of Bacterial Isolates by 16S rDNA Amplicon Sequencing

**Figure 3:**
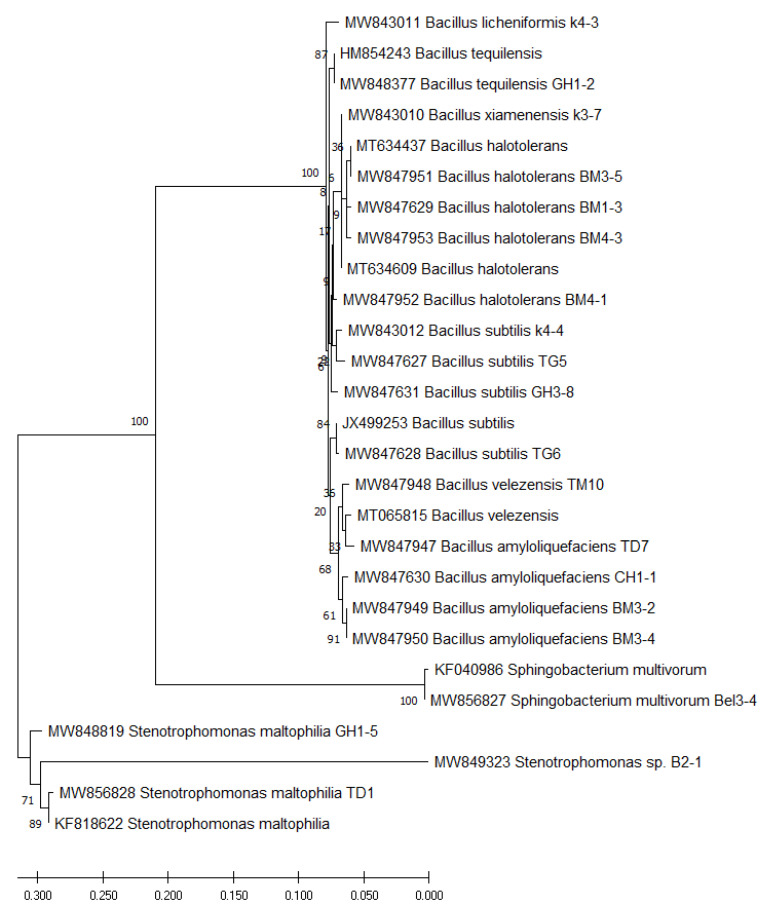
Phylogenetic tree based on maximum-likelihood analysis of nucleotide sequences of 16S rRNA gene of selected antagonist bacterial isolates against *Neocosmospora solani* using a Kimura two-parameter model in MEGA X software. The tree was evaluated via 1000 bootstrap replications.

Based on 16S rDNA sequence, the twenty rhizobacteria with an important antifungal activity against N. solani were affiliated to the genus *Bacillus* (16) (*B. subtilis* (4), *B. amyloliquefaciens* (4), *B. halotolerans* (4), *B. velezensis* (1), *B. xiamenensis* (1), *B. licheniformis* (1) and *B. tequilensis* (1)), *Stenotrophomonas* (3) (*Stenotrophomonas maltophilia* (2) and *Stenotrophomonas* sp.) and *Sphingobacterium multivorum* (1) (Figure 3).

### 2.4. Evaluation of Biocontrol Activities and Plant Growth Stimulating Traits Exercised by Selected Bacteria

#### 2.4.1. Indirect Antagonism Activity of Selected Bacteria

Volatile antifungal compounds (VOCs): The antifungal activity of bacterial VOCs against *N. solani* varied from a maximum of 48.63% (GH3-8) to a minimum of 17.97% (TG6). Results showed that four isolates K3-7, Bel3-4, GH1-8, and GH3-8 gave an inhibition rate superior to 40% (Table 2).

Antibiosis via bacterial supernatant: The antifungal activity using the bacterial cell-free culture filtrate showed that the inhibition rate was significantly dependent on the type of bacterial isolate. There were six bacterial isolates showing an inhibition rate superior to 40% (GH1-5, BM4-3, BM4-1, BM3-5, BM3-4, and B2-1) (Table 2).

#### 2.4.2. Biochemical Traits

All isolates were tested for their ability to produce cell wall-degrading enzymes 

(Figures S1 and S2; Table 3).

*Proteolytic Activity*

The results of protease production were found positive for all tested rhizobacteria (Table 3). In fact, the highest proteolytic index was registered by bacterial isolates K4-3 (2.37), K3-7 (2.13), and TD1 (2.07).

*Amylase Activity*

It was seen that all bacterial isolates were able to produce the amylase (Table 3). The three bacterial isolates TD7, BM3-5, and GH1-5 showed a high amylolytic index of 2.10, 2.07, and 1.98, respectively.

*Cellulase Degradation*

Results showed that among twenty bacterial isolates tested, sixteen had the ability to produce the cellulase (Table 3). The three bacterial isolates TD7, BM4-3, and TG5 displayed higher cellulolytic index of 2.62, 2.62, and 2.43, respectively.

*Pectinase Activity*

Results pointed out that of twenty bacterial isolates tested, eighteen had the ability to produce the pectinase (Table 3). The five bacterial isolates k3-7, BM3-5, GH1-2, and TM10 had a high pectinolytic index of 2.70, 2.57, 2.22, 2.12, and 2.04, respectively.

*Phosphate Solubilization*

The phosphate solubilization test showed that among twenty rhizobacterial isolates tested, fourteen had the ability to produce clear zones (clear halo > 1 mm) of phosphate solubilization on PVK agar medium around the colony on the plate after four days of incubation (Table 3). The highest solubilization index was obtained with three bacterial isolates GH3-8 (2.19), TD1 (2.15), and Bel3-4 (2.14).

*Indole-3-acetic Acid Production*

The results of IAA production were found positive for ten of twenty bacterial isolates and for which the supernatant culture changed to red color after the addition of the Salkowski’s reagent.

*Siderophores Production*

Results evinced that only three bacterial isolates k4-4, Bel3-4, and GH1-2 were able to produce siderophores. The change in the color of CAS from blue to orange or purple explained by the transfer of ferric ions from it to the siderophores.

*Production of Hydrogen Cyanide (HCN)*

Hydrogen cyanide (HCN), the production of which is determined by the change of Whatman paper color from the yellow to orange or brown, were produced mainly by six bacterial isolates k4-3, k4-4, B2-1, BM3-2, BM3-4, and GH1-2.

*Detection of the Antibiotic Biosynthetic Genes*

**Figure 4:**
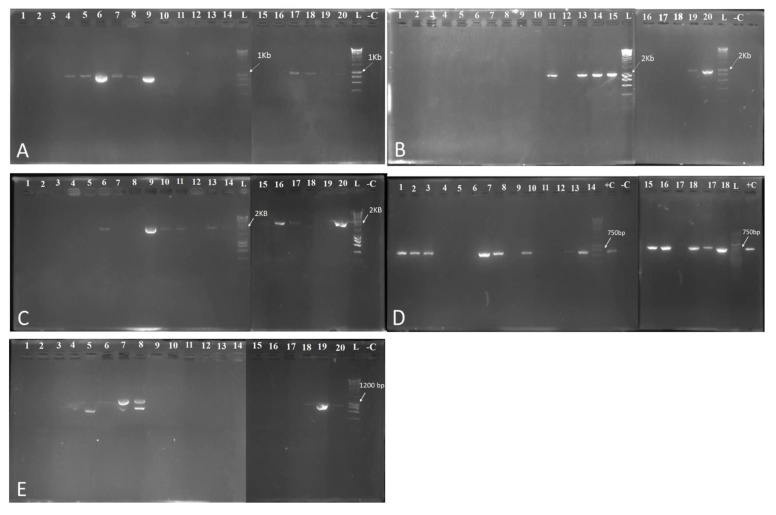
PCR detection of genes, involved in antibiotics biosynthesis, in antagonist bacteria. (**A**) bacillomycin, (**B**) fengycin, (**C**) iturin, (**D**) surfactin, and (**E**) subtilosin A. Lane 1 to lane 20: k3-7, k4-3, k4-4, Bel3-4, TD1, TD7, TG5, TG6, TM10, B2-1, BM1-3, BM3-2, BM3-4, BM3-5, BM4-1, BM4-3, GH1-1, GH1-2, GH1-5, and GH3-8, respectively; lane –C, negative control; +C, positive control; lane L, ladder.

PCR was used to investigate the biocontrol genes in the twenty selected antagonist bacteria. Results for the detection of the biosynthesis lipopeptides genes bacillomycin, fengycin, iturin, and surfactin are summarized in Figure 4. The ability of bacterial isolates to produce bacillomycin production was evaluated and results, demonstrated that only five bacterial isolates Bel3-4, TD1, TD7, TG5, TG6, TM10, GH1-1, GH1-2, GH1-5, and GH3-8 had bamC gene (band of 875 bp), which is involved in the synthesis of bacillomycin (Figure 4A). For iturin production detection, the results revealed the presence of the excepted PCR product (band of 2 Kb) in ten bacterial isolates TD7, TM10, B2-1, BM1-3, BM3-4, BM3-5, BM4-1, BM4-3, GH1-1, and GH3-8 (Figure 4B). Regarding the fengycin secretion, the results indicated that only six bacterial isolates had this gene (BM1-3, BM3-4, BM3-5 BM4-1, GH1-5, and GH3-8) (Figure 4C). However, for the surfactin production, results underlined that only six bacterial isolates lack this gene (Bel3-4, TD1, TD7, TM10, BM1-3, BM3-2, and GH1-1) (Figure 4D). Moreover, our findings pointed out that bacterial isolates TD1, GH1-5, and GH3-8 had the ability to produce subtilosin, however, the amplicons were slightly higher than the expected in TG5, while two bands are produced for bacterial isolateTG6 (Figure 4E).

### 2.5. Hypersensitivity Test

By comparing the tobacco leaves with the positive control (leaf injected with the bacterial suspension of *Agrobacterium tumefaciens*), our results showed that no bacterial isolates have induced a hypersensitivity reaction.

### 2.6. Effect of Rhizobacteria on Plant Growth of Brassica Napus

The selected bacteria were evaluated subsequently for their ability to form a beneficial association with canola by promoting its growth. Statistical analysis showed highly significant difference (*p* < 0.001) between treated and untreated canola seedlings with bacterial isolates for the plant length, root length, fresh root, and dry root weight. Interestingly, significant difference was also observed between antagonist bacteria. However, no significant difference was observed for the number of leaves. Our results showed that some rhizobacteria has a positive effect on the plant growth (Figure S3). In fact, canola seedlings treated with BM3-2, K3-7, TG5, B2-1, and K4-4 exhibited the highest increase in plant length, while those with B2-1, TD7, BM3-4, and bel3-4 showed an increase in root length. However, plants treated with TD7, BM3-2, B2-1, and Bel3-4 displayed a significant increase in total fresh weight, while those with B2-1, TM10, bel3-4, TD7 expressed a substantial increase in total dry weight. For fresh and dry root weight, only plants treated with bacterial isolates BM4-1, TD7, GH1-2, and K4-4 have registered a significant increase compared to untreated control plants (Figure S3; Table 4).

### 2.7. In Planta Bioassays

**Figure 5:**
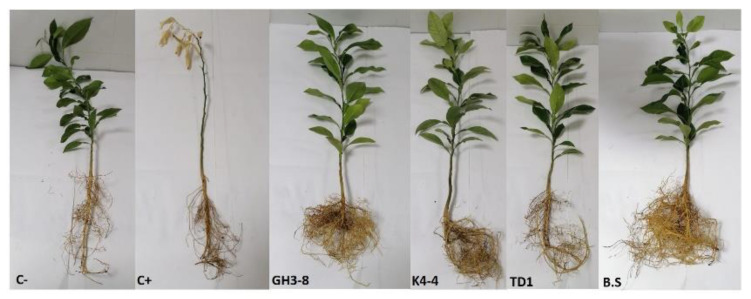
Citrus dry root rot disease symptom severity on young sour orange seedlings treated with bacterial isolates GH3-8, K4-4, TD1, and *Bacillus subtilis* commercial product and inoculated with *Neocosmospora solani* after 60 days under greenhouse conditions. C+: positive control (plants inoculated only with *N. solani*) and C−: negative control (plants received only water).

**Figure 6:**
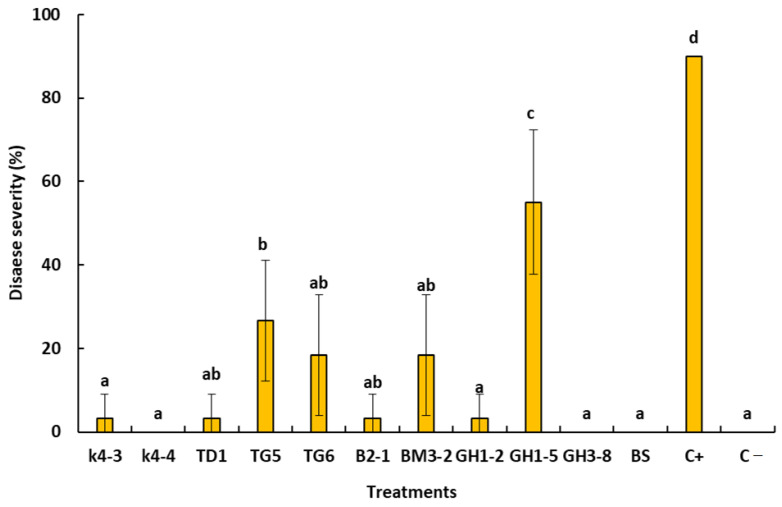
Disease severity observed on sour orange seedlings treated with bacterial isolates GH3-8, K4-4, TD1, and *Bacillus subtilis* commercial product (BS) and inoculated with *Neocosmospora solani* (1 × 10^6^ conidia/mL), after incubation at 25 °C under greenhouse conditions. C+, positive control (plants inoculated only with *N. solani*) and C−, negative control (plants received only water). Histograms represent mean value of disease severity. Error bars represent standard error and letters (a, b, c, and d) denote significant difference according to Duncan’s test (*p* < 0.05) in plant severity.

Based on the in vitro bioassay, ten antagonist bacteria were subjected to greenhouse trials to confirm their antifungal activity and their ability to suppress the citrus dry root rot disease. After eight weeks of post-inoculation periods, results indicated that bacterial isolate K4-4, GH3-8, and K4-4 were highly effective in suppressing the DRR disease (absence of disease symptoms). These bacterial isolates gave comparable results to that obtained with B. subtilis commercial product and negative control (C−) (without *N. solani*) (Figure 5 and Figure 6). Interestingly, bacterial isolates K4-3, TD1, TG6, B2-1, and BM3-2 significantly reduced the disease severity without suppressing it completely. However, bothbacterial isolates TG5 and GH1-5 were shown to be less effective in controlling the disease (Figure 6).

## 3. Discussion

In this study, we highlight that the plant rhizosphere is a valuable source of potent rhizobacteria that may serve as an eco-friendly solution for the control of soilborne diseases. In this context, a collection of 210 rhizobacteria were recovered from the citrus rhizospherre and screened for their inhibitory effect based on the in vitro dual culture bioassay between these bacterial isolates and *N. solani*. Twenty selected isolates have shown an important antifungal activity against *N. solani*. These bacterial isolates were also tested on other three other *Fusarium* species previously isolated and reported to be associated with the DRR disease [1]. Furthermore, microscopic examinations of *N. solani* mycelium in the presence of antagonist bacteria revealed the presence of structural changes including deformations, swelling, and vacuolation of the mycelium sometimes accompanied by degradation of the mycelium and release of cytoplasmic contents. The inhibition of the growth of the pathogenic fungus and damages observed on its hyphae are probably due to secretion of hydrolytic enzymes and lipopeptides by antagonist bacteria [38,39].

Previous studies have revealed the importance of antagonist bacteria in the control of *Fusarium* spp. [22,24,25,40,41,42,43]. In our study, effective bacterial isolates were identified on the basis of the partial 16S rDNA genes. Three distinct genus were distinguished namely *Bacillus, Stenotrophomonas* and *Sphingobacterium*. The association of different species with citrus rhizosphere is linked to the diversity found in the citrus root exudates that may attract these species and favour their growth in order to colonise citrus root tissues [24,44]. Over time, several bacteria are emerging as novel PGPR and declared to have biological control potentials against different fungal plant diseases. *Bacillus* species were reported in several studies to have an important potential as BCAs [45,46,47]. As our study highlighted several species belonging to the same genus, Ali et al. [47] also reported the great ability of *Bacillus tequilensis* S5 to reduce the mycelial growth by 76.6%. Indeed, the cell-free filtrates of this bacterium lowered the growth of the pathogenic fungus by 82.2%. Chenniappan et al. [44] selected sixteen bacterial isolates capable of reducing the growth of several fungal pathogens of the *Neocosmospora* and *Fusarium* genus, in particular *N. solani*. These bacterial isolates were identified as *Bacillus amyloliquefaciens*, *B. tequilensis*, and *B. subtilis.* Authors have also identified the same genes involved in the mechanisms of biocontrol as we reported in our study. *B. xiamenensis* was recently shown to have antagonistic and PGP activities and could suppress red rot disease and enhance sugarcane growth [48]. Similarly, *B. licheniformis* was previously reported as a promising BCAs against plant pathogens [49,50,51]. *B. velezensis* was previously identified to the *B. amyloliquefaciens* group [52]. Damasceno et al. [53] underscored the high performance of *B. velezensis* to reduce the mycelial growth of *Colletotrichum musae*. This bacterium was able to mitigate the same performance as the fungicidal product thiabendazole. However, Rojas-Solís et al. [54] underlined a higher antagonist activity of *Stenotrophomonas maltophilia* CR71 against *Botrytis cinerea*, which was probably realted to its ability to emit volatile organic compounds (VOCs). Moreover, this bacterium was able to promote growth and achieve an effective biocontrol of *B. cinerea* through the production of potent volatiles such as DMDS. It was found that *S. maltophilia* lowered the mycelial growth of *Colletotrichum nymphaeae* by 60% [55]. For the antagonist *Sphingobacterium multivorum*, few studies have demonstrated its capabilities as BCAs [56]. Surprisingly, our study found that *S. multivorum* inhibited the mycelial growth of *N. solani* and reduced the DRR severity. This bacterium was previously reported to have important antifungal effect against *Magnaporthe oryzae*, the causal agent of rice blast [56]. This bacterial species was also found effective in degrading hexaconazole, thereby presenting a sustainable microbial bioremediation of persistent organic pollutants [57]. *Bacillus* spp. can produce several lytic enzymes and synthesise a wide range of metabolites including biosurfactants such as lipopeptides that have the ability to be effective in controlling different plant diseases [58]. The antibiosis via bacterial supernatant indicated that the twenty rhizobacterial isolates might be a pool of various secondary metabolites. Similar results were reported by Li et al. [38] who confirmed that cell-free filtrate from *Bacillus megaterium* inhibited the mycelial growth of *Alternaria alternata.* Bacterial antagonism by antibiosis appears to be the main mechanism involved in the biological control of plant pathogenic fungi [40], in addition to the production volatile antifungal compounds (VOCs) [59]. In this study, our findings indicated that effective bacterial isolates were able to produce VOCs and inhibit mycelial growth of *N. solani*. Similarly, Guevara-Avendaño et al. [60] highlighted that organic compounds (VOCs) emitted by *B. amyloliquefaciens* were found to inhibit *N. solani* mycelial growth and induced slight distortions of fungal hyphae. VOCs are chemical substances that can be easily evaporated into the air due to their low molecular weight, high vapor pressure, and low water solubility [61,62].

In this study, the effectiveness of selected bacterial isolates to control the disease *in planta* and their performance to promote plant growth were assessed. This feature is highly suitable for BCAs and helps to suppress the disease and promote plant growth and crops productivity. In this study, the above-mentioned mechanisms were considered as an important criterion for the selection of effective PGPR. Additionally, our results emphasized that selected antagonist rhizobacteria with high antifungal activity shared excellent attributes. Most of these bacterial isolates exerted different biocontrol mechanisms such as production of cell wall degrading enzymes, production of hydrocyanic acid (HCN), along with plant growth promotion traits. Production of lytic enzymes is among the major mechanisms employed by biocontrol agents to control fungal pathogens [40,44]. Our results showed that of twenty antagonist bacterial, eighteen displayed pectinase production, while cellulase and chitinase production was observed for sixteen and eight bacteria, respectively. Interestingly, all bacteria were able to produce amylase and protease. chitinases attack fungal cell wall and cause lysis by degrading chitin [40]. In addition to hydrolytic enzymes, PGPR produce chemical compounds with different benefits for the plant. Furthermore, HCN is an antimicrobial compound synthesized from glycine using HCN synthase, an enzyme encoded by a set of three genes (hcnA, hcnB, and hcnC) [63] and it is known to be deleterious to microorganisms [40]. According to Blume et al. [64], this metabolite acts on the cells of phytopathogenic fungi by blocking cytochrome oxidase in the respiratory chain. Our results indicated that four effective bacterial isolates produced HCN. Similar results were found for *Pseudomonas fluorescens*, which produce siderophores, HCN, and chitinase, and by which the antagonist more likely impedes the growth of *Sclerotinia sclerotiorum* on *Brassica campestris* [65]. Furthermore, promising bacterial isolates were tested in vitro for properties that are known to be important for promoting plant growth, such as solubilization of phosphate, the production of siderophores, and IAA production [23]. Siderophores were characterized by a strong affinity for iron and is one of the important mechanisms used by BCAs [66,67]. Among the twenty rhizobacterial antagonists tested in this study, three isolates were siderophores producers.

The phosphates solubilizing microorganisms (PSM) are considered as potential bioinoculants to increase crop productivity by transforming phosphorus of the soil (the monobasic (H_2_PO_4_^−^) and diabetic (HPO_4_^2−^) into soluble forms assimilable by plants, through the production of organic acids, the acidification process and chelation [68]. In this study, 14 isolates were found able to solubilize tricalcium phosphate. Sun et al. [69] found that out of nineteen bacteria, thirteen were able to solubilize phosphate and ten produced IAA. Phytohormones are known to play a key role in regulating the growth of all parts of the plant during its various development stages. PGPRs can produce phytohormones, similar to those produced by plants, which enhance its growth. Additionally, they are important in generating plant defence responses against invading pathogens [41]. IAA produced by PGPRs contribute in increasing root surface area and length, and thereby better soil nutrients uptake [42,68]. Interestingly, half of effective bacterial isolates produced different shades of pinkish-red, suggesting the production of this phytohormone. Results from this study demonstrated that rhizobacteria could be effective in controlling *Neocosmospora* and *Fusarium* spp. and promote plant growth, which is in agreement with other previous studies. Our finding corroborated those of Majeed et al. [70] who found that *Stenotrophomonas* and *Bacillus* produced IAA and solubilize phosphate. A *Bacillus* sp. strain was found to strongly inhibit the in vitro growth of several phytopathogens including *N. solani* and *F. oxysporum* [71]. This bacterial isolate possessed several biocontrol and PGP mechanisms such as production of lytic enzymes, IAA, and siderophores, as well as the ability to solubilize various sources of organic and inorganic phosphates [71]. The results appeared to be the same as the report of Paramanandham et al. [25] who underscored that *Pseudomonas aeruginosa* produced IAA, HCN, siderophores, chitinase, proteases, and cellulase which played a crucial role in the antifungal activity against *F. oxysporum* f. sp. *lycopersici*, and *A. solani* on tomatoes. This result was similar to the findings of Chenniappan et al. [44] who isolated 16 antagonist bacteria from turmeric rhizosphere with the ability to produce lytic enzymes and having PGPR attributes. Our findings corroborated those of Slama et al. [22] who confirmed that *B. halotolerans* produces lytic enzymes; amylase, protease, cellulase, and chitinase, auxin, siderophores production, and phosphate solubilization that can used as BCA against Bayoud disease caused by *F. oxysporum* f. sp. *albedinis* and an efficient bio-fertilizer of oasis ecosystems. In a similar study, Palmieri et al. [41] screened four antagonist bacteria *Rhanella aquatilis*, *Pseudomonas fluorescens*, *Serratia marcescens*, and *B. amyloliquefaciens* against *Foc* and *Fsp* of wilt and root rot causing chickpea and have proved their complementary capacities to solubilize tricalcium phosphate, chitinase production, and IAA production. Our result was also in accordance with those reported by Prajakta et al. [72] who reported that *Bacillus mojavensis* PB-35(R11) exhibited phosphate solubilization, hydrogen cyanide, chitinase, IAA, and siderophores production, which may be involved in antifungal activity against *Rhizoctonia solani* and in promoting plant growth of soybean. Lim et al. [73] recently reported a *B. velezensis* strain capable of producing antifungal volatile and agar-diffusible metabolites that inhibited mycelial growth of several plant pathogenic fungi. Similarly, Martínez-Raudales et al. [74] evidenced an antifungal activity of *B. velezensis* against *N. solani*, *F. oxysporum*, *Phytophthora capsici*, and *R. solani*, causative agents of chili pepper root rot.

In this study, the biosynthesis genes encoding for the production of lipopeptides (bacillomycin, iturin, fengycin, and surfactin) and bacteriocin (subtilosin) were investigated. Surprisingly, our selected antagonist bacteria harbour biocontrol genes responsible for the production of the lipopeptides and bacteriocin. These molecules are capable of decreasing pathogen growth [75]. Therefore, our study provided valid proof confirming that genes encoding for lipopeptides played an important role in fighting against *N. solani*. The ability of bacteria to produce lipopeptides is crucial in evaluating its potential as a BCAS against plant pathogens [76]. Lipopeptides from the fengycin, surfactin, and iturin families have been shown in several studies to have considerable potential for controlling plant pathogenic fungi [44,76]. Zhang et al. [43] reported the presence of genes encoding for lipopeptides biosynthesis in most of the *Bacillus* isolates tested. Fengycins have fungitoxic properties, particularly against filamentous fungi [77]. Subtilosin A was reported to be produced by *B. amyloliquefaciens* [36]. PGPR produce a variety of antibiotic compounds including lipopeptides, which are considered as major contributors to *Bacillus* antifungal activity [78]. Cao et al. [78] demonstrated that iturin and fengycin secreted by *B. velezensis* are responsible for its antimicrobial activity, while surfactin proposed to contribute in the formation of biofilms and cell motility, both of which are important for successful rhizosphere colonization. *Bacillus velezensis* was found capable to produce a variety of antibiotics compounds including surfactin, iturin, fengycin, ericin, and others [79,80]. Zalila-Kolsi et al. [81] denoted that *B. amyloliquefaciens* and *B. subtilis* that produce (iturin and surfactin), (surfactin and fengycin), respectively, have a wide range of action against several phytopathogenic fungi according to Gong et al. [39], both iturin A and plipastatin (fengycine) A have fungicidal activity, but iturin A is active at lower concentrations than plipastatin A. In addition, treatments with both molecules showed a deformed and damaged hyphae morphology [39]. Tora et al. [33] demonstrated that the biocontrol of *Botrytis cinerea* by *Bacillus* XT1 was facilitated by lipopeptides, suggesting that mycelial structure of *B. cinerea* was probably degenerated due to these compounds.

The present study highlighted that most effective bacterial isolates were able to exhibit more than PGP traits. Our results underlined an increase in the plant growth of *B. napus* seedlings treated with antagonist bacteria (bacterization) in comparison with untreated controls. Previous studies documented the existence of multiple biocontrol mechanisms among the studied bacteria that explains their potential as successful biocontrol [44,82]. Sun et al. [69] reported that bacteria isolated from the rhizosphere enhance the plant growth of *B. napus*, which was also confirmed by the findings of Syed-Ab-Rahman et al. [83] who found that the bacterial isolates tested have contributed to a significant increase in lettuce seedling length, with the largest increase being observed for the plant treated *B. amyloliquefaciens*.

The reduction of disease severity by antagonist bacteria is more likely to be linked to their mechanisms of biocontrol and to their adaptation in the host plant environment. It has been proven that *B. subtilis* has a suppressive effect on plant pathogenic fungi under both in vitro and *in planta* conditions [84]. Treatment of cassava with *B. amyloliquefaciens* decreased the disease incidence of Fusarium root rot by more than 50% [85]. Additionally, this bacterium was found significant in suppressing the incidence of Fusarium wilt in tomato caused by *Fusarium oxysporum* f. sp. *lycopersici* [85]. Parikh et al. [86] underscored that *Bacillus* spp. suppressed the mycelial growth of pathogenic *Fusarium* isolates and decreased Fusarium root rot disease in corn, soybean, and wheat. These results were explained by the ability of *Bacillus* spp. to produce volatile organic compounds (VOCs) resulting in mycelial growth reduction of *Fusarium* spp. and causing morphological alterations in fungal hyphae [59]. Abd-Elgawad et al. [87] highlighted that the application of *B. subtilis* and *P. fluorescens* against Fusarium dry root rot of citrus reduced the incidence of the disease in treated trees and increased the yield. Application of *B. amyloliquefaciens* and *Microbacterium imperiale* against Fusarium root rot in cassava resulted in a reduction of disease incidence of more than 50% in greenhouse trials [85]. This is more likely due to the ability of this bacterium to sporulate, in addition to its higher population stability, which facilitates its storage, encapsulation, and subsequent application in the field. Zhang et al. [43] emphasized that the antagonistic and plant-growth promotion activities of bacterial isolates; *P. fluorescens*, *Pseudomonas* sp., *B. subtilis*, and *Paenibacillus polymyxa* might be related to their production of several types of lytic enzymes (β-1,3-glucanases, chitinases, and proteases), antibiotics, phytohormones (IAA), secondary metabolites, HCN, siderophores, and VOCs, and their abilities to solubilize the phosphate as well.

Practical output of our study suggests that *B. subtilis* K4-4 and Gh3-8 provided best control of DRR and could be used as BCAs due to their antifungal activity and plant growth promoting traits. Therefore, our finding represents an added value for biological control of plant diseases using antagonist bacteria; the selected bacterial isolates have a great biocontrol potential due to a higher ability of inhibiting the fungal growth and reducing the disease. Moreover, *Bacillus* species were the basis of the BCAs framework with numerous reports of their potential. PGPRs are widely used since they represent promoting traits for plant growth and they did not represent any risks to human health [88]. Our results represent an important contribution for better understanding of the citrus rhizosphere biodiversity and highlight the importance of using microbiome rhizosphere to promote plant growth and fight plant pathogens.

## 4. Materials and Methods

### 4.1. Origin of Fungal Isolates

The plant pathogenic fungi used in the present study were *Neocosmospora* (*Fusarium*) *solani* (MH999444), *F. oxysporum* (MH999445), *F. equiseti* (MH999443), and *F. brachygibbosum* (MH999442). These fungal species were previously isolated from symptomatic roots of citrus trees during the growing season 2017 in Morocco [1]. Each fungal species was subcultured on Potato Dextrose Agar (PDA) supplemented with antibiotics (Chloramphenicol and streptomycin sulfate both at 50 µg/mL) medium and incubated 7 days in darkness at 25 °C prior to experiments.

### 4.2. Isolation of Bacteria from Citrus Rhizosphere

**Figure 7:**
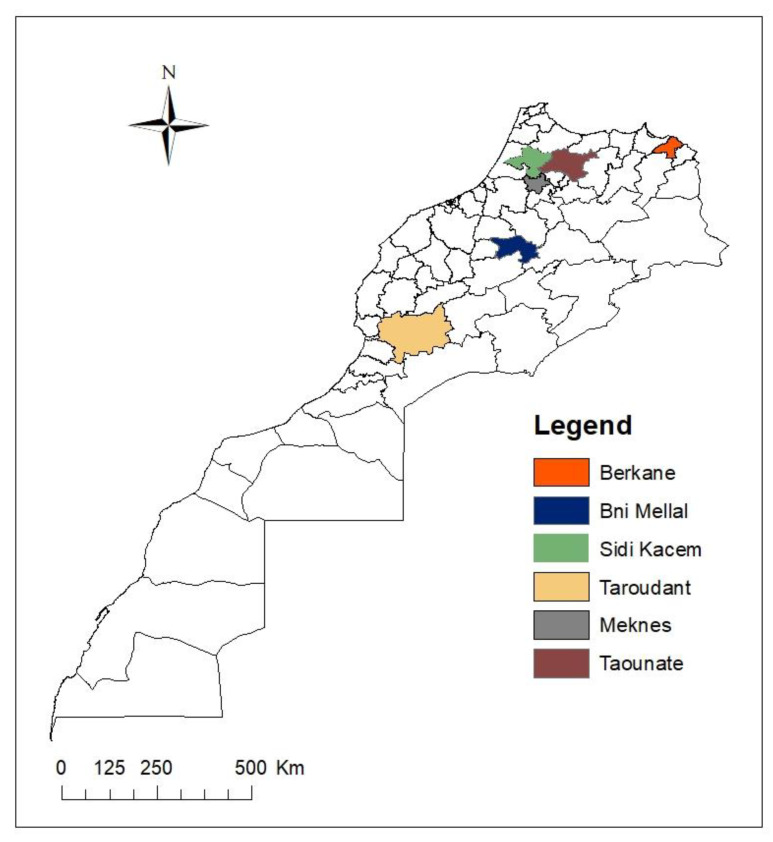
Map showing sampling fields of rhizosphere soil examined for screening of biological control agents against Citrus dry root rot disease, prepared using ArcGIS software 10.3.

The soil samples were randomly collected from citricultural areas from five regions of Morocco (Taroudant, Meknes, Taounate, Sidi Kacem, and Berkane) (Figure 7). Samples of rhizosphere soil samples with citrus roots were carefully collected. Thereafter, serial dilutions were prepared; 10 g of the soil from each sample is suspended in an Erlenmeyer flask containing 100 mL of sterile distilled water (SDW). After stirring for 30 min, resulting dilutions were spread on petri dishes containing PDA medium and then incubated at 28 °C for 72-96 h [89]. Colonies with different morphologies were selected and re-streaked on Luria Bertani medium (LB) until pure cultures are obtained. A total of 210 colonies were collected and used in subsequent screenings.

### 4.3. Screening of Antagonist Bacteria

The bacterial collection of citrus rhizosphere soil was tested in this study. The antagonism bioassay using dual culture technique by direct confrontation between bacterial isolates and fungal pathogens to screen the suitable antagonist with substantial capacity of restricting the growth of *N. solani* on PDA medium. Each bacterial isolate was streaked at four equidistant streaks along the perimeter of the Petri dish. Then, a 7 mm diameter mycelial plug was taken from the edge of a 7 day-old-colony of *N. solani* and deposited onto the center of the agar plate containing PDA medium between different streaks of each bacterium. Plates containing only the mycelial plug served as controls. The plates were sealed with parafilm and incubated at 28 °C for 7 days. The presence or absence of the inhibition zone was subsequently noted by calculating the inhibition rate of mycelial growth (IR) after one week of incubation. IR was calculated using the following formula: IR (%) = (C − T)/C × 100 With: IR: inhibition rate; C: Diameter of the fungal colony in the control plates; T: Diameter of the fungal colony in the presence of the antagonist [30].

### 4.4. Effect of Bacterial Isolates on the Mycelial Structure of Neocosmospora Solani

Interactions between the pathogenic fungus and bacterial isolates were investigated. After one week of co-culture isolates, a part of the mycelium was taken from the zone of inhibition and observed under light microscope (Ceti Microscope) to reveal the existing hyphal damages or cytological changes caused by the antagonistic bacteria compared to the control.

### 4.5. Bacterial Identification

The twenty bacteria showed to be effective against *N. solani* in the in vitro bioassay were identified by molecular tools. The genomic DNA of the bacteria was extracted using the protocol described by Llop et al. [90]. The partial 16S rDNA genes of the genomic DNA of antagonist isolates were amplified using universal primers: FD1: 5′AGAGTTTGATCCTGGCT CAG 3′ and RP2: 5′ GGTTACCTTGTTACGACTT 3’ [91]. The PCR reaction mixture was performed in a total volume of 25 μL containing 5µL of PCR buffer (5x), 1 µL (10 µM) of each primer, and 0.2 µL (5 U/µL) of Bioline taq DNA polymerase (Bioline, London, UK) and 2,5 µL of DNA template, the rest of the volume was completed with SDW. The following cycling conditions were used: Initial denaturation at 96 °C for 4 min, followed by 35 cycles of denaturation at 96 °C for 10 s, then annealing at 52 °C for 40 s and 72 °C for 2 min, and finally an extension at 72 °C for 4 min in Thermal Cycler. PCR products were sequenced in both directions using sanger sequencing method. The obtained sequences were assembled using DNAMAN software (version 6.0, Lynnon Biosoft, Quebec Canada), and compared with other bacterial DNA sequences in the National Centre for Biotechnology Information’s (National Center for Biotechnology Information (http://blast.ncbi.nlm.nih.gov/Blast.cgi). Partial sequences of 16S rDNA were deposited in the Genbank under accession numbers listed in Table 1.

### 4.6. Evaluation of Biocontrol Activities and Plant Growth Stimulating Attributes

The mechanisms of biocontrol activity and plant growth promoting traits exercised by the selected twenty rhizobacterial isolates were investigated by various in vitro experiments including production of lytic enzymes, antibiotic metabolites, HCN siderophores, IAA, and phosphate solubilization.

#### 4.6.1. Indirect Antagonist Activity of Selected Bacteria

Volatile antifungal compounds (VOCs): The ability of bacterial isolates to produce volatile VOCs against *N. solani* was investigated. Each bacterium was first inoculated in three streaks on LB medium for 24 h. Then, the lid of each plate was replaced with the bottom of another plate containing the PDA medium, inoculated with a 7 mm fresh mycelial plug of the pathogen. Subsequently, both the bottoms were sealed with transparent adhesive tape (Parafilm^®^) to prevent any loss of volatile substances [24,92].

The control was prepared in the same way except that the bottom contained no bacterium. Incubation takes place at 25 °C and the inhibition rates were noted after 7 days of incubation according to the following formula: IR= (C − T)/C × 100.

Antibiosis via bacterial supernatant: Antibiosis via bacterial supernatant was carried out by incorporation of metabolites produced by bacteria in order to assess the involvement of diffusible substances in antifungal activity [38]. An aliquote (100 μL) of each bacterial suspension (1 × 10^8^ CFU/mL) was inoculated into nutrient broth medium (NB), then incubated in a rotatory shaker at 30 °C for 3 days (130 r/min). The mixture was centrifuged for 25 min (5500 rpm) and the supernatant obtained from each isolate was filtered through a 0.22 μm diameter Millipore filter. The bacterial cell-free filtrate was incorporated into a PDA agar culture medium (45–50 °C) at a concentration of 10% (*v*/*v*). A 7 mm mycelial plug of the pathogen obtained from an actively growing culture of a 7 day-old colony was placed at the centre of the plates and further incubated at 25 °C and observations noted after 7 days of incubation. Control plates were prepared by adding a 10% concentration of liquid NB medium to the PDA instead of the bacterial supernatant. The inhibition rate was calculated as described above. There were two independent trials with 3 replicates.

#### 4.6.2. Microbial Traits

*Proteolytic activity*: The ability of antagonist bacteria to produce protease was determined using a solid medium based on skim milk. The medium was inoculated with a 5 µL (1×10^8^ CFU/mL) of each bacterial suspension. Plates were incubated at 28 °C for 48 h. Protease activity was revealed by the development of a clear halo around the colonies [83]. The proteolytic index was then calculated as the diameter of halo (mm) + diameter of a colony (mm)/diameter of a colony (mm) as described by Syed-Ab-Rahman et al. [83].

*Amylase activity*: The ability of bacterial isolates to produce amylase was assessed using a solid medium supplemented with soluble starch [93]. 5 μL of each bacterial culture (1×10^8^ CFU/mL) was spotted in the Petri dish and incubated at 28 °C for 72 h. In order to reveal the hydrolysis of the starch, the agar surface was covered with 3 mL of the iodine solution. After 3 min, the appearance of a clear zone around the colony indicated a positive amylase activity. In the absence of amylase activity, starch turns a blackish blue color. Thus, the amylolytic index is calculated as previously described [83].

*Cellulase degradation*: The bacterial antagonists were tested for their ability to produce cellulase using a solid medium supplemented with carboxymethyl cellulose (CMC). The medium was inoculated with a 5 µL (1 × 10^8^ CFU/mL) of each bacterial suspension. Plates were incubated at 28 °C for 72 h. Cellulase production was revealed by pouring Red Congo solution at a concentration of 0.1% on the surface of the Petri dishes for15 min and destaining with a solution of NaCl (1M) (by rinsing 3 times) [83]. Development of a clear halo around the colonies confirm the presence of cellulase activity, while the color remains red in the absence of cellulase activity. The cellulose index was calculated as previously described [83].

*Pectinase activity*: The ability of bacterial isolates to produce pectinases was determined using a solid medium containing pectin as previously described by Etesami et al. [94]. The medium was inoculated with a 5 µL of each bacterial suspension (1 × 10^8^ CFU/mL). The Petri dishes were incubated at 28 °C for 72 h. The pectinolytic activity was revealed by the addition of Cetyl Trimethylammonium Bromide (CTAB) at 1%. The formation of a clear halo around the colonies indicated positive pectinolytic activity. Thus, the pectinolytic index was calculated as described above.

*Chitinase activity*: Colloidal chitin amended medium (CCA) [95] was prepared to screen the ability of isobacterial lates to produce chitinase. The medium consisted of (g/L): Na2HPO4, 2; KH2PO4, 1; NH4Cl, 1; NaCl, 0.5; MgSO4 7H2O, 0.5; CaCl2 2H2O, 0.5; yeast extract, 0.5; agar, 15 and 5 g colloidal chitin [96]. Ten μL of each bacterial suspension (1 × 10^8^ CFU /mL) was inoculated on CCA medium and incubated at 28 °C for 4 days. The appearance of clear zone on CCA plates was indicative of positive results for chitinase production and the chitinolytic index was calculated using the same formula as described above.

*Phosphate Solubilization*: Pikovskaya (PVK) medium amended with 5 g/l of tricalcium phosphate (Ca3 (PO4) 2), as the sole source of phosphate, was used to test the ability of bacterial isolates to solubilize inorganic phosphate, as previously described [22,83]. The medium was inoculated with a 5 µL (1 × 10^8^ CFU/mL) of each bacterial suspension. Inoculated plates were incubated at 28 °C for 4 days. Bacterium capable of solubilizing phosphate will be surrounded by a clear halo, thus the phosphate solubilization index was calculated as described above.

*Siderophores Production***:** Siderophores production was determined using chrome azurol S (CAS-shuttle) assay [40,97] with slight modifications. Each bacterial suspension (1×10^8^ CFU/mL) was placed into 15 mL falcon tubes containing 10 mL of the liquid AT minimal medium devoid of iron [98]. After 96 h of incubation at 28 °C with stirring at 150 rpm, 0.5 mL of the supernatant was mixed with 0.5 mL of the CAS-HDTMA solution. The solution of CAS-HDTMA was prepared as follows: The ferric ion solution was freshly prepared by mixing 1.5 mL FeCl3. 6H2O (1 mM) with HCl (10 mM) and 7.5 mL of Chrome Azurol S (2 mM), stirred slowly with the addition of 6 mL hexadecyl trimethyl ammonium bromide (HDTMA), (10 mM) and shaken, the piperazine solution (4.307g of piperazine dissolved in 30 mL of water and 6.25 of HCl (12 M); pH = 5.6) was slowly added and made up to 100 mL. A tube containing 0.5 mL of the uninoculated minimal medium broth mixed with 0.5 mL of CAS-HDTMA served as a control. After 2 h of contact between the supernatant and the CAS-HDTMA solution, the blue coloring turns orange or purple when siderophores are produced.

*IAA Production*: The production of indole-3-acetic acid (IAA) was determined by the colorimetric method as described by Yuttavanichakul et al. [60]. An aliquote (100 μL) of each bacterial suspension were cultured in a liquid LB medium supplemented with L-tryptophan (1 g/L) and incubated at 28 °C on a rotatory shaker at 150 rpm for 4 days. Cultures were centrifuged at 5000 rpm for 20 min. Subsequently, 1 mL of the cell-free culture supernatant was mixed with 2 mL of Salkowski's reagent (12 g of FeCl3 per litre of 7.9 M H2SO4) and development of color was observed. The appearance of a pinkish-red coloration indicates IAA production, while yellow coloration indicates a negative result [40,88].

*Production of Hydrogen Cyanide (HCN)*: The ability of bacterial isolates to produce hydrocyanic acid (HCN) was examined following the protocol described by Lahlali et al. [30]. 100 μL of each bacterial suspension (1 × 10^8^ CFU/mL) were spread on LPGA medium supplemented with 4.4 g glycine per litre (4.4 g/L). Subsequently, sterile Whatman filter paper (no.1) discs were saturated with a picrate solution (2.5% picric acid in 12.5% anhydrous sodium carbonate (Na_2_CO_3_) solution) were placed on the lid of the Petri dish. The control plates were inoculated with SDW. The plates were sealed with parafilm and incubated at 28 °C for 4 days. The change of color from yellow to orange, red, or reddish brown indicated volatile HCN production [30].

*Detection of Lipopeptides by the PCR Method*: The total genomic DNA extracted from the twenty selected rhizobacteria were used for the detection of the biosynthesis lipopeptides (bacillomycin, fengycin, iturin, and surfactin) and bacteriocin (subtilosin A) genes. Each PCR amplifications were performed in a total volume of 25 μL of PCR mixture containing 5μL of PCR buffer (5×), 1 μL of each primer (10 μM), 0.25 μL of Taq DNA polymerase (5 U/μL) (Bioline, London, UK), 2.5 μL of genomic DNA, the rest of the volume was completed with SDW. Specific primers used for amplification of these gene was used (Table S1). PCR reactions were performed Thermal Cycler. PCR products were then visualized on a 1.5% agarose gel colored using cyber safe (Invitrogen, CA, USA) by electrophoresis and visualized with an ultraviolet illuminator and digitally recorded.

### 4.7. Greenhouse Experiment

#### 4.7.1. Hypersensitivity Test

The phytopathogenicity of the twenty antagonist bacteria was examined using the hypersensitivity test on tobacco (*Nicotiana tabacum*). An aliquote (100 μL) of each bacterial suspension (1 × 10^8^ CFU/mL) was injected at the level of the midrib of the lower part of the leaf. For the positive and negative controls, a bacterial suspension of a plant pathogen (*Agrobacterium tumefaciens*) and SDW were used, respectively. The treated plants were kept in a growth chamber at a temperature of 27 °C and observed after 24 to 48 h. A phytopathogenic bacterium elicits a positive hypersensitivity response that results in leaf dryness and brown necrosis.

#### 4.7.2. PGPR Test on *Brassica Napus*

The twenty effective bacterial isolates (IR > 60%) were tested for their PGPR capabilities *in planta*. Bacterial isolates were cultured in yeast extract peptone (YEP) medium at 28 °C with shaking. After 24 h of incubation, the cell cultures were centrifuged at 5000 rpm for 20 min, the supernatant was removed, and the pellet was suspended in phosphate buffered saline (PBS) at a concentration of 1 × 10^8^ CFU/mL (OD = 0.1 to λ = 600 nm). *Brassica napus* was used due to its fast growth, large biomass production, and high germination rate [99,100]. Canola seeds, with uniform shape and size, used in this trial were surface sterilized with 95% ethanol for 30 s, washed with SDW 5 times, and air dried at room temperature. Subsequently, they were soaked in the bacterial culture diluted in PBS for 1 h at room temperature. Seeds soaked in the PBS solution were served as a control.

The soil was sterilized twice by autocalving at 121 °C for 90 min to destroy the microorganisms it contains. Subsequently, 140 g of soil was placed in pots (20 cm × 7 cm) previously disinfected with sodium hypochlorite and dried at ambient temperature. One seed was planted per pot and pots were arranged in complete randomized design with 5 replications. This trial was carried out in a controlled greenhouse at a temperature of 25 °C. After 30 days, three pots from each treatment were randomly selected and the plants/roots length and the plants/roots fresh/dry weight were measured and recorded [69,83].

#### 4.7.3. In Planta Antagonism

Based on the in vitro results, ten isolates (k4-4, TD1, TG5, TG6, B2-1, BM3-2, GH1-2, GH1-5, and GH3-8) from different studied regions, that suppressed the growth of *N. solani* more than 60% and exhibit several biocontrol mechanisms were selected for greenhouse experiment.

The chosen bacteria were grown on YPGA broth with shaking at 150 rpm for 48 h at room temperature, centrifuged and resuspended in PBS. The concentration of cells was approximately 1 × 10^9^ CFU/mL (OD 600 = 0.8–1) and used as bacterial inoculum. The fungal inoculum was prepared as described by Ezrari et al. [1] and Freitas et al. [85] with some modifications. Fungal conidial suspension was prepared by adding 5 mL of sterile saline buffer (0.85 % *w*/*v* NaCl) to each Petri plate in order to obtain a conidial suspension which it passed through a double-layer sterile cheesecloth and the pathogen concentration was adjusted to 1 × 10^6^ conidia/mL using a hemocytometer. Sour orange seedlings 8-month old were carefully removed from their substrates, cleared from soil debris, washed, and their roots were injured before being inoculated by dipping into conidial fungal suspension for 30 min. After transplanting the inoculated plants into new pots containing a sterile soil substrate, the rest of conidial suspension was added to the pot to ensure contact of the pathogen with the roots. Afterwards, the bacterial suspension (100 µL of each isolate) was added to the pot. The inoculated seedlings were kept in a greenhouse at 25 °C. Plants were watered two to three times weekly. The experiment was conducted as follows (i) negative control (SS alone) (ii) positive control (sterilized soil (SS) + fungus), (iii) isolate K4-3 (SS + fungus + K4-3), (iv) isolate K4-4 (SS + fungus + K4-4), (v) isolate TD1 (SS + fungus + TD1), (vi) isolate TG5 (SS + fungus + TG5), (vii) isolate TG6 (SS + fungus + TG6), (viii) isolate B2-1 (SS + fungus + B2-1), (ix) isolate BM3-2 (SS + fungus+ BM3-2) (×) isolate GH1-2 (SS + fungus + GH1-2), (xi) isolate GH1-5 (SS + fungus + GH1-5), (xii), isolate GH3-8 (SS + fungus + GH3-8) and (xiii) *Bacillus subtilis* (SS + fungus + Commercial product). Plants were arranged in a randomized block with 3 replicates for each treatment. The effect of the application of bacterial inoculant on reduction of disease symptoms was assessed after 2 months. Plants were examined for disease severity through visual observations using a 1–5 scale: 1 (0%) was attributed to healthy plants, 2 (10%) indicated partially defoliated plants with interveinal chlorosis (1–20% of the foliage affected), 3 (35%) indicated partially defoliated plants with interveinal chlorosis (21–50% of the foliage affected), 4 (65%) indicated partially defoliated plants that was accompanied by leaf yellowing (51–80%) of the foliage affected), and 5 (90%) indicated plants displaying total defoliation.

### 4.8. Statistical Analysis

All in vitro and *in planta* experiments were carried out twice over time. All tests were carried out using a completely randomized design. All datasets were summarized as mean ± SD (standard deviation). The Arcsin transformation was applied to assess the disease severity. All statistical analysis were performed using SPSS statistical software (version 20, IBM SPSS Statistics 20, New York, NY, USA) and when the effect was revealed to be significant, Duncan test was performed for means separation at a significance level of *p* ≤ 0.05.

## 5. Conclusions

In the present study, research of effective biocontrol agents for the control of dry root rot of citrus, was started by in vitro screening of rhizobacteria. In light of the results obtained, twenty bacteria were selected to have an important antifungal activity against *N. solani* and tested also against three other *Fusarium* species associated with this disease. The selected bacteria were studied for their biochemical characteristic and evaluated for their abilities to enhance the growth of *B. napus* under greenhouse conditions. Ten isolates were also tested for their capacity to control the DRR disease under greenhouse conditions. Two bacteria appear promising since they were found to completely suppress the disease. These antagonist bacteria, isolated from the soil of healthy citrus, fulfil the hypothesis according to which microorganisms chosen as BCAs should be screened from their local niches, and used in the same environment in order to obtain the desired benefits. In addition, their plant growth traits were sought as possible additional mechanisms. Undoubtedly, the encouraging results of this study are important and open new alternatives toward the design of biocontrol strategies for managing DRR and limit losses in citrus crops. Therefore, two antagonists bacteria *B. subtilis* K4-4 and GH3-8 were proposed to control and prevent damages of DRR disease. However, to confirm their biocontrol potential at a large-scale, further experiments under natural conditions and during environmental stress would also be necessary through inoculations in citrus orchards and especially during stress conditions. Furthermore, the implementation of BCAs at a large-scale faces several challenges, which depends on the advances of interdisciplinary research, in particular mass production and formulation methods, which are crucial factors to have effective bacterial inoculums with higher reliability and competitiveness on the market.

## Figures and Tables

**Table 1:** The in vitro inhibition rates (%) of mycelial growth of *Neocosmospora solani*, *Fusarium oxysporum*, *F. brachygibbosum*, and *F. equiseti*, obtained with twenty selected bacterial isolates from citrus rhizosphere.

Region	Bacteria	*N. solani*	*F. oxysporum*	*F. brachygibbosum*	*F. equiseti*
Taroudant	k3-7	**64.48 ± 1.38 ^d–h^**	54.75 ± 1.67 ^d,e,f^	59.87 ± 0.58 ^e,f,g^	9.25 ± 2.13 ^d,e,f^
Taroudant	k4-3	**66.39 ± 1.81 ^h,i^**	53.02 ± 1.18 ^c,d^	53.65 ± 0.76 ^c,d^	59.44 ± 0.34 ^d,e,f^
Taroudant	k4-4	**72.22 ± 0.17 ^j^**	54.08 ± 1.73 ^c,d,e^	56.75 ± 0.96 ^d,e^	**68.73 ± 1.40 ^g^**
Sidi Kacem	Bel3-4	**65.58 ± 0.27 ^e–i^**	45.98 ± 0.51^b^	48.60 ± 1.00 ^b^	50.54 ± 1.54 ^a,b^
Taounate	TD1	**71.50 ± 1.13 ^j^**	51.53 ± 0.16 ^c,d^	57.72 ± 1.71 ^e,f^	59.12 ± 0.40 ^d,e,f^
Taounate	TD7	**63.96±1.25 ^b–h^**	47.17 ± 1.44 ^b^	**60.98 ± 1.00 ^e–h^**	52.64 ± 0.72 ^b,c^
Taounate	TG5	**65.96 ± 0.29 ^g,h,i^**	46.65 ± 0.59 ^b^	59.31 ± 0.87 ^e,f,g^	**60.25 ± 0.03 ^e,f^**
Taounate	TG6	**64.29 ± 1.25 ^c–h^**	46.10 ± 0.49 ^b^	**62.47 ± 1.51 ^g,h^**	55.99 ± 0.60 ^c,d^
Taounate	TM10	**64.07 ± 1.70 ^b–h^**	43.83 ± 0.37 ^b^	51.93 ± 1.90 ^b,c^	48.31 ± 0.77 ^a^
Berkane	B2-1	**62.63 ± 1.80 ^a–g^**	38.01 ± 1.54 ^a^	34.48 ± 1.56 ^a^	56.51 ± 1.49 ^d,e^
Bni Mellal	BM1-3	**59.16 ± 0.66 ^a^**	57.10 ± 1.46 ^e,f,g^	**67.89 ± 0.38 ^j,k^**	**62.66 ± 0.64 ^f^**
Bni Mellal	BM3-2	**65.87 ± 0.44 ^f–i^**	57.95 ±1.26 ^f,g^	**70.59 ± 1.18 ^k,l^**	**60.65 ±0.13 ^f^**
Bni Mellal	BM3-4	**63.24 ± 0.29 ^b–g^**	59.75 ± 1.62 ^g,h^	**66.87 ± 1.93 ^i,j,k^**	**69.29 ± 0.70 ^g,h^**
Bni Mellal	BM3-5	**60.84 ± 0.10 ^a–d^**	**67.62 ± 0.57 ^j^**	**67.01 ± 1.69 ^i,j,k^**	**66.35 ± 1.10 ^g^**
Bni Mellal	BM4-1	**64.36 ± 0.28 ^d–h^**	**75.42 ± 0.75 ^k^**	**64.41 ± 1.28 ^h,i,j^**	**68.92 ± 1.68 ^g^**
Bni Mellal	BM4-3	**60.63 ± 1.54 ^a,b,c^**	**68.21 ± 0.29 ^j^**	**63.11 ± 1.16 ^g,h,i^**	**69.76 ± 1.77 ^g,h^**
Sidi Kacem	GH1-1	**62.22 ± 1.14 ^a–f^**	**63.21± 0.93 ^i^**	**72.12 ± 0.72 ^l^**	**72.81 ± 1.00 ^i^**
Sidi Kacem	GH1-2	**61.89 ± 1.67 ^a–e^**	51.18 ± 1.60 ^c^	59.94 ± 1.66 ^e,f,g^	59.97 ± 1.54 ^e,f^
Sidi Kacem	GH1-5	**60.49 ± 0.90 ^a,b^**	57.52 ± 0.36 ^e,f,g^	57.74 ± 1.80 ^e,f^	59.19 ± 1.50 ^d,e,f^
Sidi Kacem	GH3-8	**68.93 ± 0.53 ^i,j^**	**62.13 ± 1.17 ^h,i^**	**61.51 ± 0.97 ^f,g,h^**	**61.04 ± 1.22 ^f^**

Data represent mean ± standard deviation (SD). Values having the same letter, in the same column, are not significantly diferent according to the Duncan test (*p* < 0.05).

**Table 2:** Effect of bacterial cell-free culture filtrates (at 10% *v/v*) and Volatile antifungal compounds (VOCs) on the in vitro mycelial growth of *Neocosmospora solani* after 7 days of incubation at 25 °C.

Bacterial Isolate Code	Species	Accession Numbers	Cell–Free Filtrates	VOCs
k3-7	*B. xiamenensis*	MW843010	29.93 ± 0.65 ^a,b^	43.70 ± 0.86 ^i^
k4-3	*B. licheniformis*	MW843011	31.83 ± 1.16 ^b,c^	35.03 ±1.02 ^e^
k4-4	*B. subtilis*	MW843012	35.17 ± 1.55 ^d,e^	37.31 ± 0.97 ^e,f,g^
Bel3-4	*S. multivorum*	MW856827	30.89 ± 0.02 ^b^	43.82 ± 0.77 ^i^
TD1	*S. maltophilia*	MW856828	27.92 ± 0.23 ^a^	36.61 ± 0.93 ^e,f^
TD7	*B. amyloliquefaciens*	MW847947	32.02 ± 1.21 ^b,c^	20.79 ± 1.00 ^a,b^
TG5	*B. subtilis*	MW847627	32.36 ± 0.46 ^b,c^	22.85 ± 0.63 ^b,c^
TG6	*B. subtilis*	MW847628	37.63 ± 0.50 ^e,f,g^	17.97 ± 0.70 ^a^
TM10	*B. velezensis*	MW847948	34.13 ± 1.12 ^c,d^	39.85 ± 1.15 ^g,h^
B2-1	*Stenotrophomona* sp.	MW849323	40.70 ± 0.36 ^h,i,j^	24.79 ± 1.15 ^c,d^
BM1-3	*B. halotolerans*	MW847629	39.39 ± 1.31 ^g,h,i^	38.92 ± 0.58 ^f,g,h^
BM3-2	*B. amyloliquefaciens*	MW847949	36.63 ± 0.31 ^d,e,f^	25.40 ± 0.57 ^c,d^
BM3-4	*B. amyloliquefaciens*	MW847950	43.65 ± 0.35 ^k^	27.29 ± 0.56 ^d^
BM3-5	*B. halotolerans*	MW847951	40.69 ± 0.20 ^h,i,j^	19.10 ± 1.88 ^a^
BM4-1	*B. halotolerans*	MW847952	41.82 ± 0.91^i,j,k^	20.25 ± 0.65 ^a,b^
BM4-3	*B. halotolerans*	MW847953	42.35 ± 0.34 ^j,k^	36.70 ± 1.44 ^e,f^
CH1-1	*B. amyloliquefaciens*	MW847630	39.30 ±0.15 ^g,h,i^	25.12 ±1.07 ^c,d^
GH1-2	*B. tequilensis*	MW848377	38.71 ±0.41 ^f,g,h^	27.70 ±0.55 ^d^
GH1-5	*S. maltophilia*	MW848819	41.91 ± 0.22 ^i,j,k^	41.10 ± 0.42 ^h,i^
GH3-8	*B. subtilis*	MW847631	36.05 ± 1.65 ^d,e^	48.63 ± 0.56 ^j^

Data represent mean ± standard deviation (SD). Values having the same letter, in the same column, are not significantly diferent according to the Duncan test (*p* < 0.05).

**Table 3:** Ability of twenty selected bacterial antagonists to produce lytic enzymes and Promoting growth plants (PGP) traits involved in the biocontrol mechanisms such as pectinase, protease, amylase, cellulose, chitinase, phosphate solubilization and hydrocyanic acid (HCN), IAA, and siderophore production.

Isolates	PI	PrI	AI	CI	ChI	PSI	HCN	IAA	SD
k3-7	2.70 ± 0.05 ^l^	2.13 ± 0.02 ^d^	1.24 ± 0.09 ^c^	2.16 ± 0.05 ^d,e^	1.00 ± 0.00 ^a^	2.11 ± 0.09 ^a^	−	−	−
k4-3	1.89 ± 0.02 ^g^	2.37 ± 0.29 ^e^	1.64 ± 0.05 ^f,g^	2.22 ± 0.04 ^d,e^	1.00 ± 0.00 ^a^	1.00 ± 0.00 ^f,g,h^	+	−	−
k4-4	1.67 ± 0.05 ^f^	1.42 ± 0.09 ^b,c^	1.86 ± 0.01 ^I,j^	2.08 ± 0.04 ^d^	1.00 ± 0.00 ^a^	2.05 ± 0.03 ^f^	+	−	+
Bel3-4	1.46 ± 0.03 ^c,d,e^	1.49 ± 0.14 ^c^	1.45 ± 0.01 ^d^	2.16 ± 0.03 ^d,e^	1.00 ± 0.00 ^a^	2.14 ± 0.08 ^g,h^	−	−	+
TD1	1.42 ± 0.08 ^c^	2.07 ± 0.19 ^d^	1.55 ± 0.02 ^e^	2.11 ± 0.03 ^d^	1.85 ± 0.35 ^e^	2.15 ± 0.09 ^g,h^	−	−	−
TD7	1.42 ± 0.02 ^c^	1.33 ± 0.14 ^a,b,c^	2.10 ±0.08 ^l^	2.62 ± 0.28 ^f^	1.00 ± 0.00 ^a^	1.00 ± 0.00 ^a^	−	+	−
TG5	1.43 ± 0.04 ^c,d^	1.28 ± 0.15 ^a,b,c^	1.69 ± 0.03 ^g,h^	2.43 ± 0.21 ^e,f^	2.01 ± 0.32 ^e^	1.00 ± 0.00 ^a^	−	+	−
TG6	1.00 ± 0.00 ^a^	1.15 ± 0.04 ^a^	1.71 ± 0.08 ^g,h^	1.00 ± 0.00 ^a^	1.00 ± 0.00 ^a^	1.28 ± 0.06 ^d^	−	+	−
TM10	2.04 ± 0.01 ^h^	1.31 ± 0.06 ^a,b,c^	1.66 ± 0.03 ^g,h^	1.00 ± 0.00 ^a^	1.00 ± 0.00 ^a^	1.19 ± 0.04 ^c^	−	+	−
B2-1	1.29 ± 0.03 ^b^	1.39 ± 0.07 ^b,c^	1.81 ± 0.04 ^i^	1.00 ± 0.00 ^a^	1.08 ± 0.03 ^a,b^	1.44 ± 0.05 ^e^	+	−	−
BM1-3	1.00 ± 0.00 ^a^	1.31 ± 0.13 ^a,b,c^	1.92 ± 0.08 ^j,k^	1.47 ± 0.02 ^b^	1.00 ± 0.00 ^a^	1.14 ± 0.03 ^b,c^	−	−	−
BM3-2	1.24 ± 0.03 ^b^	1.34 ± 0.08 ^a,b,c^	1.73 ± 0.03 ^h^	1.55 ± 0.00 ^b,c^	1.00 ± 0.00 ^a^	2.14 ± 0.03 ^g,h^	+	+	−
BM3-4	1.50 ± 0.05 ^d,e^	1.31 ± 0.09 ^a,b,c^	1.58 ± 0.00 ^e,f^	2.23 ± 0.09 ^d,e^	1.41 ± 0.15 ^c^	1.15 ± 0.06 ^c^	+	−	−
BM3-5	2.57 ± 0.03 ^k^	1.25 ± 0.11 ^a,b^	2.07 ± 0.05 ^l^	2.17 ± 0.24 ^d,e^	1.65 ± 0.27 ^d^	1.14 ± 0.04 ^b,c^	−	−	−
BM4-1	1.52 ± 0.04 ^e^	1.22 ± 0.07 ^a,b^	1.16 ± 0.02 ^b^	2.29 ± 0.32 ^d,e^	1.27 ± 0.11 ^b,c^	1.00 ± 0.00 ^a^	−	−	−
BM4-3	2.22 ± 0.02 ^j^	1.19 ± 0.03 ^a,b^	1.25 ± 0.04 ^c^	2.62 ± 0.23 ^f^	1.00 ± 0.00 ^a^	1.07 ± 0.03 ^a,b^	−	+	−
GH1-1	1.46 ± 0.03 ^c,d,e^	1.30 ± 0.11 ^a,b,c^	1.03 ± 0.01 ^a^	1.77 ± 0.28 ^c^	1.00 ± 0.00 ^a^	1.00 ± 0.00 ^a^	−	−	−
GH1-2	2.12 ± 0.01 ^i^	1.28 ± 0.03 ^a,b,c^	1.53 ± 0.01 ^e^	2.09 ± 0.01 ^d^	1.00 ± 0.00 ^a^	2.09 ± 0.01 ^f,g^	+	+	+
GH1-5	1.60 ± 0.05 ^f^	1.42 ± 0.14 ^b,c^	1.98 ± 0.02 ^k^	1.00 ± 0.00 ^a^	1.19 ± 0.05 ^a,b^	1.00 ± 0.00 ^a^	−	+	−
GH3-8	1.24 ± 0.03 ^b^	1.26 ± 0.07 ^a,b,c^	1.71 ± 0.06 ^g,h^	2.20 ±0.03 ^d,e^	1.97 ± 0.28 ^e^	2.19 ± 0.06 ^h^	−	+	−

PI: pectinolytic index, PrI: proteolytic index, AI: amylolytic index, CI: cellulosic index, ChI: chitinolytic index, PSI: phosphate solubilizing index; HCN: hydrocyanic acid, IAA: Indole-3-acetic acid, SD: siderophore (+): positive reaction; (−): negative reaction. All index were calculated as the diameter of halo (mm) + diameter of a colony (mm)/diameter of a colony (mm). Data represent mean ± standard deviation (SD). In each column, values having the same letter are not significantly different according to the Duncan’s test (*p* < 0.05).

**Table 4:** Growth attributes of *Brassica napus* seedlings treated with the twenty selected antagonist bacteria against untreated control.

Bacterial Isolate	LN	TL	RL	TFW	RFW	RDW	TDW
k3-7	4 ± 1.00 ^a^	22.67 ± 2.517 ^c,d^	15.83 ± 1.16 ^f,g,h^	1.51 ± 0.07 ^b,c,d^	0.81 ± 0.11 ^d,e,f^	0.007 ± 0.001 ^b,^^c^	0.086 ± 0.007 ^a,b^
k4-3	4 ± 0.00 ^a^	15.17 ± 0.76 ^a^	13.83 ± 1.16 ^d,e,f^	1.28 ± 0.55 ^a,b,c^	0.44 ± 0.04 ^a^	0.006 ± 0.002 ^a,b^	0.068 ± 0.013 ^a,b^
k4-4	4 ± 0.00 ^a^	21.84 ± 2.57 ^c,d^	16.00 ± 1.80 ^f,g,h^	1.69 ± 0.09 ^d,e,f,g^	0.94 ± 0.13 ^f,g^	0.013 ± 0.001 ^e,f^	0.085 ± 0.030 ^a,b^
Bel3-4	4 ± 0.00 ^a^	20.40 ± 1.44 ^b,c,d^	17.67 ± 1.53 ^h,i^	2.08 ± 0.14 ^g,h^	0.69 ± 0.14 ^b,c,d,e^	0.010 ± 0.001 ^d,e,f^	0.221 ± 0.059 ^d^
TD1	3.68 ± 0.58 ^a^	20.17 ± 2.47 ^b,c,d^	13.67 ± 1.16 ^c,d,e,f^	1.70 ± 0.15 ^d,e,f,g^	0.69 ± 0.08 ^b–e^	0.010 ± 0.001 ^d,e,f^	0.102 ± 0.015 ^b,c^
TD7	4 ± 0.00 ^a^	20.33 2.52 ^b,c,d^	18.33 ± 0.76 ^h,i^	2.56 ± 0.18 ^i^	1.10 ± 0.17 ^g^	0.012 ± 0.002 ^d,e,f^	0.19 ± 0.021 ^d^
TG5	4 ± 0.00 ^a^	22.50 ± 2.29 ^c,d^	15.83 ± 0.29 ^f,g,h^	1.94 ± 0.13 ^e,f,g,h^	0.52 ± 0.06 ^a,b^	0.009 ± 0.001 ^c,d,e^	0.089 ± 0.017 ^a,b^
TG6	4 ±1.00 ^a^	15.33 ± 1.89 ^a^	14.17 ± 1.04 ^d,e,f^	1.36 ± 0.04 ^a,b,c,d^	0.47 ± 0.05 ^a^	0.008 ± 0.001 ^b,c,d^	0.088 ± 0.016 ^a,b^
TM10	4 ± 0.00 ^a^	19.17 ± 2.57 ^a–d^	14.84 ± 1.89 ^e,f,g^	1.92 ± 0.14 ^e,f,g,h^	0.85 ± 0.13 ^e,f^	0.010 ± 0.001 ^c,d,e^	0.266 ± 0.067 ^e^
B2-1	4.33 ± 0.58 ^a^	22.50 ± 3.91 ^c,d^	19.33 ± 2.47 ^i^	2.12 ± 0.52 ^h^	0.75 ± 0.15 ^c,d,e,f^	0.011 ± 0.001 ^d,e,f^	0.269 ± 0.044 ^e^
BM1-3	3.68 ± 0.58 ^a^	15.50 ± 2.29 ^a^	12.17 ± 2.08 ^b,c,d^	1.48 ± 0.06 ^b,c,d^	0.56 ± 0.11 ^a,b,c^	0.011 ± 0.002 ^d,e,f^	0.090 ± 0.011 ^a,b^
BM3-2	4 ± 0.00 ^a^	23.10 ± 1.91 ^d^	13.17 ±1.06 ^c,d,e^	2.13 ± 0.09 ^h^	0.90 ± 0.15 ^e,f^	0.011 ± 0.002 ^d,e,f^	0.146 ± 0.025 ^c^
BM3-4	4.33 ± 0.58 ^a^	17.60 ± 1.40 ^a,b^	18.17 ± 1.76 ^h,i^	1.64 ± 0.14 ^c,d,e,f^	0.64 ± 0.08 ^a,b,c,d^	0.005 ± 0.002 ^b,c^	0.070 ± 0.008 ^a,b^
BM3-5	3.33 ± 0.58 ^a^	18.67 ± 2.31 ^a,b,c^	12.03 ± 1.62 ^a,b,c,d^	1.51 ± 0.05 ^b,c,d^	0.580 ± 0.09 ^a,b,c^	0.006 ± 0.003 ^b,c^	0.087 ± 0.014 ^a,b^
BM4-1	4.33 ± 0.58 ^a^	16.67 ± 1.53 ^a,b^	16.83 ± 1.04 ^g,h,i^	1.94 ± 0.06 ^e,f,g,h^	1.11 ± 0.13 ^g^	0.013 ± 0.001 ^f^	0.048 ± 0.010 ^a^
BM4-3	4 ± 0.00 ^a^	15.17 ± 0.76 ^a^	14.33 ± 1.04 ^d,e,f,g^	1.19 ± 0.06 ^a,b^	0.56 ± 0.03 ^a,b,c^	0.007 ± 0.005 ^b,c^	0.090 ± 0.014 ^a,b^
GH1-1	4.33 ±0.58 ^a^	16.93 ± 2.53 ^a,b^	11.90 ± 0.96 ^a,b,c,d^	1.49 ± 0.33 ^b,c,d^	0.76 ± 0.07 ^c,d,e,f^	0.010 ± 0.002 ^c,d,e^	0.142 ± 0.013 ^c^
GH1-2	4 ± 1.00 ^a^	17.50 ± 1.32 ^a,b^	11.17 ± 0.76 ^a,b,c^	1.57 ± 0.19 ^b,c,d,e^	0.95 ± 0.07 ^f,g^	0.012 ± 0.001 ^e,f^	0.103 ± 0.010 ^b,c^
GH1-5	4 ± 0.00 ^a^	17.00 ± 1.32 ^a,b^	12.17 ± 1.26 ^b,c,d^	1.98 ± 0.10 ^f,g,h^	0.86 ± 0.18 ^e,f^	0.010 ± 0.002 ^c,d,e^	0.093 ± 0.023 ^a,b^
GH3-8	4 ± 0.00 ^a^	16.00 ±2.65 ^a^	9.57 ±0.95 ^a^	1.44 ± 0.131 ^a,b,c,d^	0.70 ± 0.03 ^b,c,d,e^	0.010 ± 0.002 ^d,e,f^	0.107 ± 0.008 ^b,c^
Untreated control	3 ± 0.00 ^a^	15.60 ± 0.40 ^a^	10.50 ± 1.00 ^a,b^	1.08 ± 0.06 ^a^	0.73 ± 0.15 ^b,c,d,e^	0.003 ± 0.001 ^a^	0.080 ± 0.004 ^a,b^

Data represent mean ± standard deviation (SD). In each colmun, values having the same letter are not signficantly different according to Duncan test (*p* < 0.05). LN: Leaves number; TL: Total length; RL: Root lenght, TFW: Total fresh weight; RFW: Root fresh weight, RDW: Root dry weight, TDW: Total dry weight.

## Data Availability

Data is contained within the article.

## Supplementary Materials

The following are available online at https://www.mdpi.com/article/10.3390/plants10050872/s1, Figure S1: Detection of various enzymatic and antagonistic traits of representative bacterial isolates, Figure S2: ndole-3-acetic acid (IAA) and Siderophores production by representative antagonistic bacterial isolates, Figure S3: Effect of inoculation with rhizobacterial isolates (Right) on Brassica napus growth compared to control (Left), 30 days after sowing, Table S1: Oligonucleotide sequences of lipopeptides and bacteriocin genes.
