# Supplementary material for: Potential Role of Rhizobacteria Isolated from Citrus Rhizosphere for Biological Control of Citrus Dry Root Rot
Source: Plants (Basel). 2021 Apr 26;10(5):872. doi: 10.3390/plants10050872 (PMC8145030; doi:10.3390/plants10050872)
Supplement: Supplementary file 1 [file plants-10-00872-s001.zip › plants-1192378-supplementary.pdf]

plants MDPI

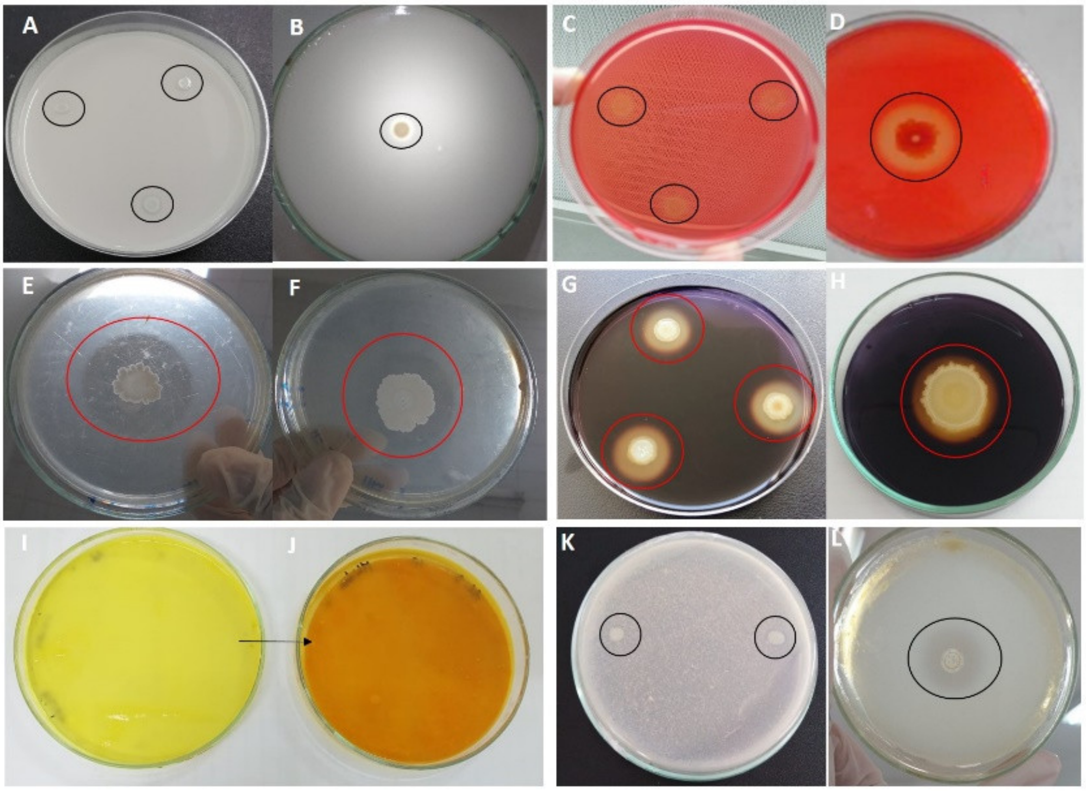

Figure S1. Detection of various enzymatic and antagonistic traits of representative bacterial isolates. A-B: Phosphate tricalcium solubilization, C-D: Cellulase activity, E-F: Protease activity, G-H: Amylase activity, I: HCN production, K: Chitinase activity and H: colloidal chitin degradation.

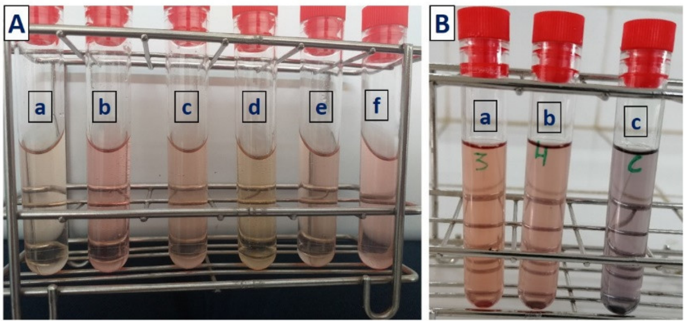

Figure S2. Indole-3-acetic acid (IAA) and siderophores production by representative antagonistic bacterial isolates. (A) IAA with a: B2-1, b: TM10, c: TG6, d: control with no bacteria, e: TG5 and f: TD7; (B) Siderophores with a: k4-4 and b: Bel3-4 and c: control with no bacteria.

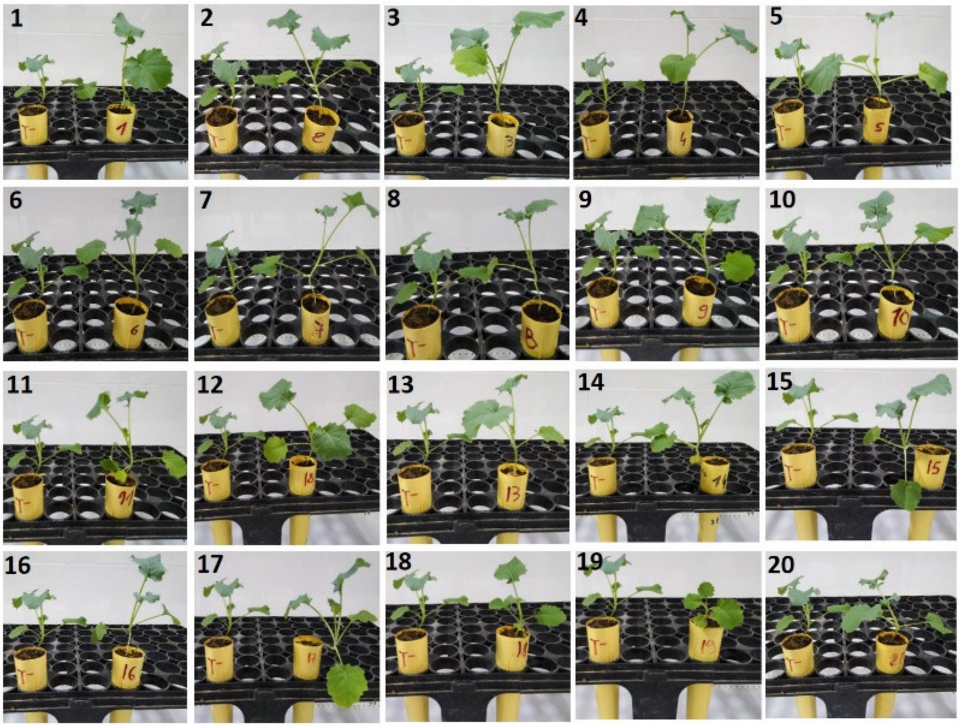

Figure S3. Effect of inoculation with rhizobacterial isolates (Right) on *Brassica napus* growth compared to control (Left), 30 days after sowing. 1 to 20: k3-7, k4-3, k4-4, Bel3-4, TD1, TD7, TG5, TG6, TM10, B2-1, BM1-3, BM3-2, BM3-4, BM3-5, BM4-1, BM4-3, GH1-1, GH1-2, GH1-5 and GH3-8 respectively.

Table S1. Oligonucleotide sequences of lipopeptides and bacteriocin genes

Lipopeptides	Design Primer	Primer sequence	Product length (bp)	Annealing T°	References
Bacillomycin	Bacc1F /Bacc1R	GAAGGACACGGCAGAGAGTC/ CGCTGATGACTGTTCATGCT	875 bp	60 °C	[76]
Fengycin	Fend1F/ Fend1R	TTTGGCAGCAGGAGAAGTT/ GCTGTCCGTTCTGCTTTTTC	964 bp	62 °C	[76]
Iturin	Itup1F/ Ituo2R	AGCTTAGGGAACAATT-GTCATCGGGGCTTC/ TCAGATAGGCCGCCATATCG-GAATGATTCG	2 kb	45 °C	[77]
Surfactin	P17/ P18	ATGAAGATTTACGGAATTTA/ TTATAAAAGCTCTTCGTACG	675 bp	53 °C	[78]
Subtilosin	OsboP1N/ OsboP2N	CCTCATGACCAG-GACTTCGCCTT/ CGGTGCCGAGCGCTTCAGGT	1200 bp	58 °C	[79]
